# The Cerebellum Is a Common Key for Visuospatial Execution and Attention in Parkinson’s Disease

**DOI:** 10.3390/diagnostics11061042

**Published:** 2021-06-06

**Authors:** Wataru Sako, Takashi Abe, Yuki Matsumoto, Kazumi Nakamura, Shotaro Haji, Yusuke Osaki, Masafumi Harada, Yuishin Izumi

**Affiliations:** 1 Department of Neurology, Tokushima University Graduate School of Biomedical Sciences, Tokushima 770-8503, Japan; kaducbfc@tokushima-u.ac.jp (K.N.); aura_shochan@hotmail.com (S.H.); yosaki@tokushima-u.ac.jp (Y.O.); yizumi@tokushima-u.ac.jp (Y.I.); 2 Department of Radiology, Tokushima University Graduate School of Biomedical Sciences, Tokushima 770-8503, Japan; abe-takashi@med.nagoya-u.ac.jp (T.A.); matsumoto.yuuki@tokushima-u.ac.jp (Y.M.); masafumi@tokushima-u.ac.jp (M.H.)

**Keywords:** Parkinson’s disease, cognition, functional MRI, network, cerebellum

## Abstract

Cognitive decline affects the clinical course in patients with Parkinson’s disease (PD) and contributes to a poor prognosis. However, little is known about the underlying network-level abnormalities associated with each cognitive domain. We aimed to identify the networks related to each cognitive domain in PD using resting-state functional magnetic resonance imaging (MRI). Forty patients with PD and 15 normal controls were enrolled. All subjects underwent MRI and the Mini-Mental State Examination. Furthermore, the cognitive function of patients with PD was assessed using the Montreal Cognitive Assessment (MoCA). We used independent component analysis of the resting-state functional MRI for functional segmentation, followed by reconstruction to identify each domain-related network, to predict scores in PD using multiple regression models. Six networks were identified, as follows: the visuospatial-executive-domain-related network (*R*^2^ = 0.54, *p* < 0.001), naming-domain-related network (*R*^2^ = 0.39, *p* < 0.001), attention-domain-related network (*R*^2^ = 0.86, *p* < 0.001), language-domain-related network (*R*^2^ = 0.64, *p* < 0.001), abstraction-related network (*R*^2^ = 0.10, *p* < 0.05), and orientation-domain-related network (*R*^2^ = 0.64, *p* < 0.001). Cerebellar lobule VII was involved in the visuospatial-executive-domain-related and attention-domain-related networks. These two domains are involved in the first three listed nonamnestic cognitive impairment in the diagnostic criteria for PD with dementia (PDD). Furthermore, Brodmann area 10 contributed most frequently to each domain-related network. Collectively, these findings suggest that cerebellar lobule VII may play a key role in cognitive impairment in nonamnestic types of PDD.

## 1. Introduction

Traditionally, a focal brain lesion was believed to cause a specific neurological sign or symptom; however, this notion has recently been challenged [1]. One possible reason is that the region implicated in a particular function depends on the specific individual. That is, the size and position of an area related to a particular function are diverse among individuals; for example, this is clearly demonstrated in aphasia [2] Another reason may be that systems, rather than regions, are responsible for each function. That is, different lesions may produce similar neurological signs or symptoms via a common network. Overlapping lesions within a network were found for several clinical syndromes using lesion network mapping [1] In line with these findings, network analysis has been extended to study neurological disorders including epilepsy [3], Parkinson’s disease (PD), [4,5], dementia with Lewy bodies [4], and Alzheimer’s disease [4] Several different approaches have been proposed to perform network analysis, such as electroencephalography, functional magnetic resonance imaging (fMRI), diffusion tensor imaging, and fluorodeoxyglucose positron emission tomography (FDG PET). Of these, fMRI is expected to provide high spatial resolution and allow the detection of functionally related regions, which are identified by their synchronous fluctuations. Independent component analysis (ICA) is a technique for analysis of resting-state fMRI. This method reveals macro-scale spatiotemporal organization, which is reproducibly composed of intrinsic connectivity networks, including the default mode network (DMN), dorsal and ventral attention networks, salience network, auditory network, visual network, and cerebellar network [6] These normal networks were assessed in PD [7,8,9,10,11,12,13], and group-level comparisons were mainly performed in a univariate, voxel-wise manner that had limitation for single-subject measurements of the network activity as a whole [14] To overcome this issue, resting-state fMRI data have been quantitated with disease-related network topographies that were used in FDG PET [14,15]

PD is recognized as a neurodegenerative disorder that is characterized by bradykinesia, rigidity, and resting tremor. Patients with PD also present with a broad spectrum of nonmotor symptoms, such as cognitive impairment, which affects the clinical course and contributes to a poor prognosis [16,17] Cognitive impairment is reported to be associated with microtubule-associated protein tau H1 haplotype [18,19]; apolipoprotein epsilon 4 alleles [20,21,22] the glucocerebrosidase gene [23,24]; and cerebrospinal fluid levels of amyloid beta 1–42 [25,26,27,28,29,30,31], alpha-synuclein [30,32], and tau [25,26,33] Network abnormalities may also be associated with cognitive impairment in PD, and univariate analysis revealed the areas related to cognitive impairment in PD, as described in the previous paragraph [7,10,11,12,13,34] In addition, one multivariate analysis demonstrated both disease- and verbal-learning-related networks for PD [14] However, although Montreal Cognitive Assessment (MoCA) is frequently used in clinical practice and recommended for patients with PD [35], network-level abnormalities related to each MoCA domain have not yet been elucidated. We therefore functionally segmented nodes by ICA to reconstruct disease- and domain-related networks beyond the existing normal networks and tested the hypothesis that a distinct network contributes to each MoCA domain in PD.

## 2. Materials and Methods

### 2.1. Subjects

We studied 40 patients with PD and 15 normal controls in Tokushima University Hospital between June 2015 and April 2019. Patients were diagnosed with PD according to the UK Brain Bank criteria [36] All subjects underwent MRI and the Mini-Mental State Examination (MMSE). The MMSE was used to define the normal controls (Ctr), who had MMSE scores ≥24 and normal activity of daily living. The MRI scan for patients with PD was obtained during the “off” period. The cognitive function of patients with PD was assessed with the Japanese version of the MoCA in detail. The levodopa equivalent dose (LED) was based on the following formula: [37] levodopa/carbidopa × 1 + entacapone × 0.35 + pramipexole × 100 + ropinirole × 20 + rotigotine × 10 + selegiline × 10 + amantadine × 1. The subjects’ characteristics are summarized in Table 1. Written informed consent was obtained from all subjects following a detailed explanation of the procedures, and the study protocol was approved by the local ethics committee of Tokushima University Hospital. The characteristics of the subjects are summarized in Table 1. 

### 2.2. MRI Acquisition

Image acquisition was completed using a 3.0 T Discovery 750 scanner (GE) at Tokushima University Hospital. The scan parameters of the resting-state fMRI included field of view (FOV) = 240 mm, matrix = 64 × 64, TR = 2000 ms, TE = 27.2 ms, flip angle = 77°, and slice thickness = 3.0 mm. The parameters of the T1-weighted images included FOV = 240 mm, matrix 256 × 256, TR = 7.77 ms, TE = 2.98 ms, flip angle = 8°, and slice thickness = 0.9 mm. All subjects were instructed to close their eyes without making any movements or thinking any specific thoughts.

### 2.3. Voxel-Based Morphometry

Voxel-based morphometry was conducted using SPM12 (http://www.fil.ion.ucl.ac.uk/spm/ (accessed on 5 June 2021)) with MATLAB version R2016b (MathWorks, Natick, MA, USA). Using the segmentation function in SPM12, structural images were segmented into three different classes: gray matter, white matter, and cerebrospinal fluid. A template was created using Diffeomorphic Anatomical Registration Through Exponentiated Lie Algebra (DARTEL). A segment of gray matter was warped to the DARTEL template in Montreal Neurological Institute (MNI) space following an initial affine registration. The images were modulated to hold information about the volume and were smoothed using a Gaussian filter with 8 mm full width at half maximum (FWHM). Group differences were considered significant at a voxel-level threshold of *p* < 0.001 (uncorrected), with a correction for cluster extent at *p* < 0.05. Furthermore, the total gray matter volume was calculated using a gray matter mask.

### 2.4. Network Analysis

To improve the study quality before analysis, we excluded any subjects who showed motion displacement of 3 mm or more, to minimize the effect of motion on MRI. Detailed methods for the network analysis were described previously [14] A principal component analysis (PCA) was performed to reduce the dimensions of the data followed by a group ICA. The number of independent components (ICs) was determined to be 72 according to the minimum description length criteria [38], and this allowed for functional segmentation [39] The InfoMax algorithm was repeated 100 times using bootstrap resampling in ICASSO [40] The cluster stability of each IC was estimated using the I_q_ index [40] ICs were assessed based on the expectation that reliable networks should present activated regions in the cortex, nucleus, or both, with time courses dominated by low-frequency fluctuations [41] Furthermore, we classified ICs into the auditory network, cognitive control network, DMN, somatomotor network, visual network, cerebellar network, subcortical network, and non-resting-state network, according to previous reports [6,39,42] ICs were excluded if their entire volume was <50 voxels when the cut-off for connectivity within each network was 1.0. Subject-level spatial networks were generated by back reconstruction through spatial–temporal regression [43] The network expression of a given group-level network for each subject was calculated as a network score using the scaled subprofile model (SSM)/PCA [14,44] Network scores were included as independent variables in a stepwise multiple regression model to predict each domain of the MoCA, and the generated model was evaluated by *R*^2^ values. This type of model-based network was defined as each domain-related network, which was expressed as a linear combination of the networks according to the estimated model coefficients.

### 2.5. Statistics

The two-tailed Student’s *t*-test was used for comparing continuous variables between groups, with a significance level set at *p* < 0.05. All statistical analyses, including the multiple regression analysis for the model-based network, were performed using SPSS Statistics version 21 (IBM, Armonk, NY, USA). 

## 3. Results

### 3.1. Voxel-Based Morphometry

Using voxelwise analysis, it was found that there were no significant differences between the normal controls and the patients with PD. In addition, there was no significant difference in the total gray matter volume between the two groups (Ctr, 603 ± 72.4 mL; PD, 608 ± 65.2 mL; *p* = 0.77). 

### 3.2. Independent Component Analysis (ICA)

**Figure 1:**
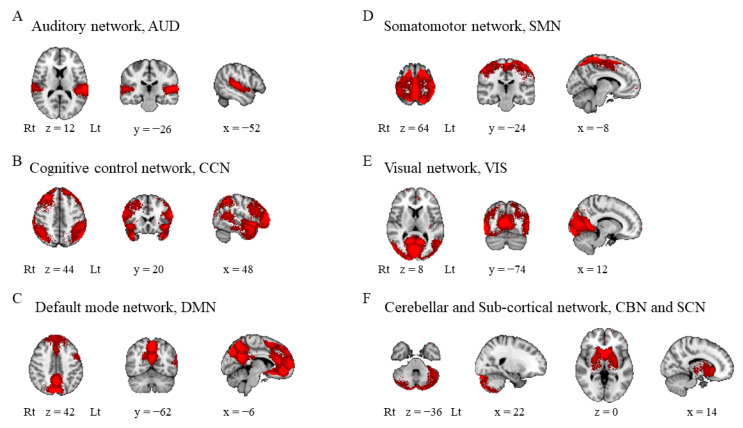
Intrinsic connectivity networks. Independent components were classified into seven networks: (**A**) auditory network, (**B**) cognitive control network, (**C**) default mode network, (**D**) somatomotor network, (**E**) visual network, and (**F**) cerebellar and subcortical networks. Lt, left; Rt, right.

No subjects were excluded because of motion displacement. We identified 72 ICs, of which 70 had an I_q_ of at least 0.8. Of these, 18 ICs were excluded because they were recognized as artifacts (IC2, IC13, IC16, IC19, IC21, IC26, IC27, IC29, IC53, and IC69) or had <50 voxels (IC32, IC41, IC52, IC55, IC58, IC68, IC70, and IC72). Finally, the ICs were classified into seven networks: [42] the auditory network (IC42; Figure 1A), cognitive control network (IC11, IC20, IC22, IC24, IC28, IC31, IC34, IC37–39, IC43–45, IC48–51, IC56, IC57, IC59, IC61, IC64, IC66, and IC71; Figure 1B), DMN (IC4, IC6, IC8, IC10, IC12, IC15, IC17, IC18, IC25, IC60, and IC67; Figure 1C), somatomotor network (IC1, IC5, IC7, IC9, IC35, IC40, and IC42; Figure 1D), visual network (IC14, IC30, IC46, IC54, IC62, and IC63; Figure 1E), cerebellar network (IC36, IC47, and IC65; Figure 1F), and subcortical network (IC3, and IC33; Figure 1F). These ICs for further analysis are summarized in Supplementary Table S1. 

### 3.3. Each Domain-Related Network

**Figure 2:**
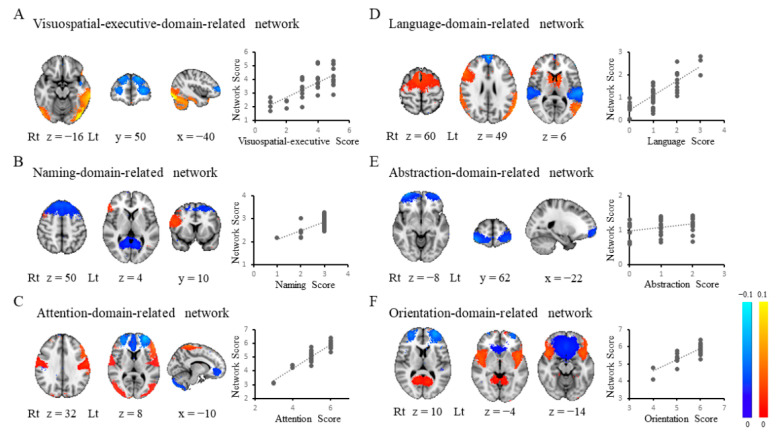
Each domain-related network. Each domain-related network was determined with a multiple regression model to explain the relationship between brain networks and each domain score: (**A**) the visuospatial executive domain, (**B**) the naming domain, (**C**) the attention domain, (**D**) the language domain, (**E**) the abstraction domain, or (**F**) the orientation domain. The cerebellum is featured in the visuospatial-executive-domain-related and attention-domain-related networks (**A**,**B**). Lt, left; Rt, right.

**Figure 3:**
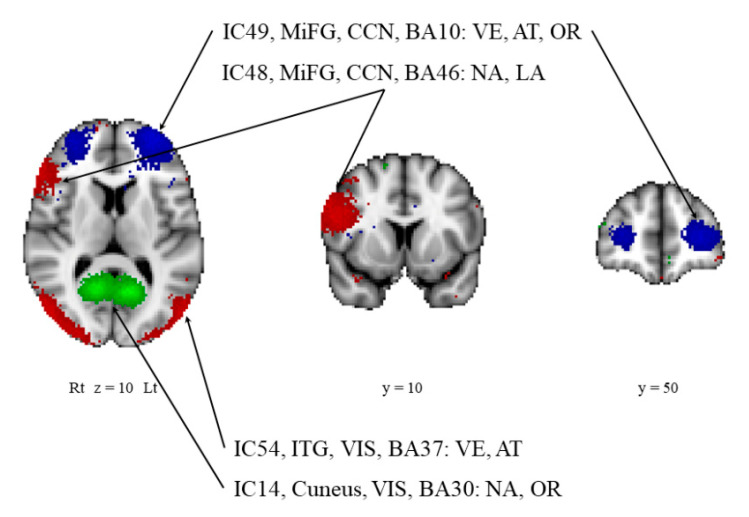
Independent components involved in multiple networks. The independent components that are involved in multiple networks are shown. Note that BA10 contributed most frequently to each domain-related network. Blue and red indicate negative and positive correlations, respectively, while green indicates either a negative or positive correlation. AT, attention-domain-related network; BA, Brodmann area; CCN, cognitive control network; IC, independent component; ITG, inferior temporal gyrus; LA, language-domain-related network; Lt, left; MiFG, middle frontal gyrus; NA, naming-domain-related network; OR, orientation-domain-related network; VE, visuospatial-executive-domain-related network, VIS, visual network.

Each domain-related network was computed from the ICs in a linear model as follows: visuospatial-executive-domain-related network (*R*^2^ = 0.54, *p* < 0.001; IC31, IC49, IC54, IC47, and IC56; BA21, BA10, BA37, cerebellar lobule VI/VII/VIII, and BA8; Figure 2A), naming-domain-related network (*R*^2^ = 0.39, *p* < 0.001; IC25, IC48, and IC14; BA8, BA46, and BA30; Figure 2B), attention-domain-related network (*R*^2^ = 0.86, *p* < 0.001; IC49, IC71, IC66, IC36, IC42, IC15, IC1, IC54, IC7, and IC22; BA10, BA38, BA46, cerebellar lobule VII, BA6, BA11, BA4, BA37, BA3, and BA38; Figure 2C), language-domain-related network (*R*^2^ = 0.64, *p* < 0.001; IC24, IC3, IC17, IC23, IC62, IC35, and IC48; BA20, caudate nucleus, BA8, BA42, BA19, BA6, and BA46; Figure 2D), abstraction-domain-related network (*R*^2^ = 0.10, *p* < 0.05; IC61; BA10; Figure 2E), orientation-domain-related network (*R*^2^ = 0.64, *p* < 0.001; IC49, IC4, IC50, IC14, and IC40; BA10, BA32, BA38, BA30, and BA6; Figure 2F). The delayed-recall-domain-related network was not generated in the present study. Of note, cerebellar lobule VII was found in the networks related to visuospatial executive or attention function. These two domains are considered to be core symptoms of dementia in PD [45] Each domain-related network is summarized in Table 2. Four ICs were found to be associated with multiple networks and are thus considered to be key hubs linking several networks (IC14, IC48, IC49, and IC54; BA30, BA46, BA10, and BA37; Figure 3). 

### 3.4. Group Differences

Decreased network scores in the PD group were observed in ICs including the anterior cingulate (IC4, *p* = 0.02), cerebellar lobule VII (IC36, *p* = 0.04), or superior temporal gyrus (IC50, *p* < 0.05), while increased network scores in the PD group were observed in ICs including lobule VI (IC47, *p* = 0.04) or the inferior frontal gyrus (IC66, *p* = 0.04). These results are shown in Supplementary Figure S1.

## 4. Discussion

### 4.1. Each Domain-Related Network without the Cerebellum

Using ICA of resting-state fMRI, we identified the networks associated with each MoCA cognitive domain such as the visuospatial-executive-domain-related network, naming-domain-related network, attention-domain-related network, language-domain-related network, abstraction-domain-related network and orientation-domain-related network.

The naming-domain-related network included BA8, BA46, and BA30, and the language-domain-related network consisted of BA20, the caudate nucleus, BA8, BA42, BA19, BA6, and BA46. The left BA46 is recognized as part of the left dorsolateral prefrontal cortex (DLPFC) and is mainly implicated in the language production system [46,47] However, this was not the case because the region of BA46 in the present study was found on the right side of the brain. A recent study reported decreased functional connectivity (FC) between the vermis and the right DLPFC in PD with cognitive impairment [48] Given these findings, the right DLPFC might therefore be involved in cognitive impairment in PD. The right DLPFC is pivotal for memory retrieval from voice [49], which might link language-domain assessments to the right DLPFC; i.e., this domain includes instructions to name something starting with one kana (Japanese alphabet) or the alphabet and to repeat two sentences after hearing them. As memory load induces hyperactivity in the right DLPFC in older people compared to younger people [50], simultaneous memories of features in pictures of animals might make this region involved in the naming-domain-related network as well as the language-domain-related network in older patients with PD. Memory load was also related to the medial frontal gyrus (BA6) [51], and working memory activated the medial frontal gyrus (BA8) [52] The caudate nucleus was reported to be the most frequently affected region related to language processing deficits after stroke [53], which supported our finding that the caudate nucleus participated in the language-domain-related network. In terms of input processing, the naming-domain-related network required BA30, a part of the visual network, while the language-domain-related network included BA42, a part of the auditory network. The former was induced by visual stimuli, while the latter was induced by auditory stimuli. These findings suggest a strict functional separation between the assessments. BA30 was also involved in the orientation-domain-related network, in addition to BA6, BA10, BA32, and BA38. The superior temporal gyrus (BA38) was related to visual–spatial orienting [54] and the attribution of intention [55], both of which can contribute to orientation.

### 4.2. Each Domain-Related Network with the Cerebellum

The visuospatial-executive-domain-related network was characterized by nodes involved in visual processing, including BA37, BA21, and cerebellar lobule VI/VII. BA37, also involved in the attention-domain-related network, is part of the ventral stream of visual processing [56,57], which is key for the performance of visuospatial tasks. BA21, activated by Japanese kana, [58], contributed to this network because kana was used in the visuospatial executive domain of the Japanese version of the MoCA, as a substitute for the alphabet. Cerebellar lobule VI was reported to functionally connect with the middle temporal visual area, a part of the dorsal stream, rather than with the ventral stream [59] Cerebellar lobule VII is widely connected with the frontal cortices [60] and may be associated with BA10, BA8, or both within this network. BA10 is hypothesized to allow the holding of goals in the mind during some tasks [61], which is based on working memory, prospective memory, and the manipulation of information to maintain and execute intended actions. Indeed, the assessment of these functions has been demonstrated to elicit activation in the BA10 [62,63,64] Intentional movement induced corticomuscular coupling in the gamma band detected by intracerebral stereo electroencephalography in the BA10, in contrast to theta band for imitative movement [65] These findings provide a convincing argument that BA10 plays a crucial role in the performance of tasks that require several steps, such as visuospatial-executive or attention tasks. The sensorimotor network appears to be incorporated into the attention-domain-related network. Attention was reported to be associated with the sensorimotor network in traumatic brain injury and PD [66,67] The superior temporal gyrus (BA38) is related to visual–spatial orienting [54] In addition, BA10, BA37, and cerebellar lobule VII, the nodes in the attention-domain-related network, overlap with the visuospatial-executive-domain-related network. Of these, the cerebellum is the most fascinating region as previous studies have demonstrated changes in cerebellar activity and connectivity measured by fMRI [14,68,69,70] FDG PET [71], and N-isopropyl-p−123-I-iodoamphetamine single-photon emission computed tomography [72] and alpha-synuclein inclusions in the cerebellum [73] Kawabata and his colleagues classified nondemented patients with PD into two types: PD with amnestic cognitive deficit (PD-A) and PD with nonamnestic cognitive deficit (PD-NA) [12] Patients with PD-NA tend to have Lewy body pathology in contrast to PD-A, because Alzheimer’s disease pathology is reportedly associated with a reduced likelihood of visual hallucinations or attentional fluctuations in dementia with Lewy bodies [74,75,76,77] FC in cerebellar lobule VII is reduced in PD-NA. Collectively, nonamnestic symptoms, visual hallucinations and attentional fluctuations, are implicated in Lewy body pathology and aberrant FC in cerebellar lobule VII, which appears to be in good agreement with our findings that cerebellar lobule VII was involved in both the visuospatial-executive-domain-related network and the attention-domain-related network. These two domains are listed as the first three nonamnestic cognitive impairments in the diagnostic criteria for PD with dementia (PDD) [45] and may be a suitable biomarker for PDD. In this line, a combination of the visuospatial-executive-domain-related network and the attention-domain-related network might be an objective biomarker for PDD with predominant Lewy body pathology. 

### 4.3. Basic-Network-Level Abnormalities

Network-level alterations for the DMN and the dorsal attention network were found in PD, PD with mild cognitive impairment (MCI), and PDD, but the results were heterogeneous among studies [7,8,9,10,11,12,13] The DMN is thought to be decreased in patients with PD and is positively correlated with cognitive scores. Indeed, whereas FC in one part of the DMN was positively correlated with verbal/visual memory and visuospatial scores [7,13,34], FC in another part of the DMN was negatively correlated with visuospatial/visuoperceptive scores as reported in the present study [10] These findings might indicate that the relationship between FC of the DMN and cognitive function was dependent on the DMN region. The part of the other networks, including the dorsal attention network, visual network, frontoparietal network, and cerebellum–brainstem network, was reportedly reduced in patients with PD [9,10,11,12] In contrast, the DMN, frontal pole network, left frontoparietal network, and cerebellar network were found to be in part increased in patients with PD [10,11] The result would be different even within the same network for the same disease if the node was different. Group ICA-based functional segmentation allows for node-level analysis [39], which revealed the presence of the following in PD: decreased FC in the anterior cingulate cortex within the DMN, in the cerebellum within the cerebellar network, and in the superior temporal gyrus within the cognitive control network; increased FC in the cerebellum within the cerebellar network and in the inferior frontal gyrus within the cognitive control network. Furthermore, this method provides the flexible integration of each node according to the hypothesis, using network scores and models. 

### 4.4. Limitations

The main limitation of this study was the lack of a validation group due to the paucity of subjects. A relatively large number of subjects is required to conduct functional segmentation. For the same reason, we were unable to perform subgroup analyses (e.g., PDD) or adjust for confounding factors. The MoCA is a screening test, and a full neuropsychological evaluation may be preferable for detecting networks related to accurately separated domains. Furthermore, our enrolled subjects were diagnosed not by pathology, but by clinical examination. In this sense, other diseases might be included in this study. 

## 5. Conclusions

The cerebellar lobule VII was identified as a common hub between the visuospatial-executive-domain-related network and the attention-domain-related network. These domains are associated with PD with nonamnestic dementia/MCI, and thus the cerebellar lobule VII might have a key role in cognitive impairment of a nonamnestic type. In contrast, amyloid beta and tau burden may contribute to a reduced likelihood of visual hallucinations and attentional fluctuations. Altogether, the two networks including cerebellar lobule VII may allow us to evaluate the predominance of Lewy body pathology over Alzheimer pathology in each patient with PD; however, the networks that include cerebellar lobule VII need to be validated in individuals who are classified by autopsy or amyloid/tau PET examination. 

## Figures and Tables

**Table 1:** Characteristics of the subjects recruited in this study.

Group	Age (Year)	Male (Female)	Handedness Right (Left)	Disease Duration (Years)	HY	MMSE	MoCA	LED
Ctr	66 ± 13.9	7 (8)	14 (1)	NA	NA	28.6 ± 1.55	NA	NA
PD	69 ± 9.0	22 (18)	36 (4)	5.1 ± 5.84	2.1 ± 0.97	26.5 ± 2.67	22.2 ± 3.56	201 ± 274.7

Abbreviations: Ctr, control; HY, Hoehn–Yahr stage; LED, levodopa equivalent dose; NA, not available; PD, Parkinson’s disease. Mean ± standard deviation.

**Table 2:** Multiple regression analysis with each subdomain as dependent variable.

Dependent Variable	*R* ^2^	Independent Variable	BA/Lobule	B	95% CI	*p* Value
visuospatial/executive	0.54	IC31, MTG, CCN	21	0.063	0.024 to 0.101	0.002
		IC49, MiFG, CCN	10	−0.060	−0.112 to −0.009	0.024
		IC54, ITG, VIS	37	0.050	0.019 to 0.082	0.003
		IC47, Cerebellum	VI, VII, VIII	0.070	0.009 to 0.132	0.026
		IC56, SFG, CCN	8	−0.058	−0.112 to −0.005	0.034
naming	0.39	IC25, SFG, DMN	8	−0.026	−0.043 to −0.010	0.003
		IC48, MiFG, CCN	46	0.030	0.008 to 0.052	0.008
		IC14, Cuneus, VIS	30	−0.012	−0.023 to −0.002	0.019
attention	0.86	IC49, MiFG, CCN	10	−0.059	−0.078 to −0.039	<0.001
		IC71, STG, CCN	38	0.056	0.036 to 0.075	<0.001
		IC66, IFG, CCN	46	0.043	0.026 to 0.059	<0.001
		IC36, Cerebellum	VII	−0.027	−0.043 to −0.011	0.002
		IC42, MiFG, SMN	6	0.028	0.016 to 0.040	<0.001
		IC15, MeFG, DMN	11	−0.031	−0.042 to −0.020	0.005
		IC1, PrG, SMN	4	0.012	0.004 to 0.020	0.005
		IC54, ITG, VIS	37	0.020	0.008 to 0.032	0.002
		IC7, PoG, SMN	3	−0.025	−0.042 to −0.008	0.005
		IC22, STG, CCN	38	−0.017	−0.032 to −0.002	0.029
language	0.64	IC24, Uncus, CCN	20	−0.119	−0.159 to −0.079	<0.001
		IC3, CN		0.035	0.017 to 0.054	0.001
		IC17, MeFG, DMN	8	−0.046	−0.067 to −0.024	<0.001
		IC23, STG, AUD	42	−0.036	−0.057 to −0.015	0.001
		IC62, MTG, VIS	19	0.054	0.009 to 0.098	0.020
		IC35, MeFG, SMN	6	0.023	0.002 to 0.044	0.034
		IC48, MiFG, CCN	46	0.033	0.001 to 0.065	0.043
abstraction	0.10	IC61, MiFG, CCN	10	−0.025	−0.049 to 0.000	0.047
delayed recall	NA	NA		NA	NA	NA
orientation	0.64	IC49, MiFG, CCN	10	−0.050	−0.068 to −0.031	<0.001
		IC4, AC, DMN	32	−0.010	−0.016 to −0.004	0.001
		IC50, STG, CCN	38	0.034	0.016 to 0.052	<0.001
		IC14, Cuneus, VIS	30	0.011	0.002 to 0.021	0.024
		IC40, MeFG, SMN	6	−0.044	−0.083 to −0.005	0.027

Abbreviations: AC, anterior cingulate; AG, angular gyrus; AUD, auditory network; BA, Brodmann area; auditory network; CCN, cognitive control network; CI, confidence interval; CN, caudate nucleus; DMN, default mode network; HY, Hoehn–Yahr stage; IFG, inferior frontal gyrus; ITG, inferior temporal gyrus; LED, levodopa equivalent dose; MeFG, medial frontal gyrus; MiFG, middle frontal gyrus; MTG, middle temporal gyrus; NA, not available; PoG, postcentral gyrus; PrG, precentral gyrus; SCN, subcortical network; SFG, superior frontal gyrus; SMN, somatomotor network; STG, superior temporal gyrus; VIS, visual network.

## Data Availability

The data presented in this study are available on request from the corresponding author. The data are not publicly available due to restrictions due to privacy issues.

## Supplementary Materials

The following are available online at https://www.mdpi.com/article/10.3390/diagnostics11061042/s1, Figure S1: Group differences in network scores, Table S1: Summary of the independent components.
