# Supplementary material for: The Cerebellum Is a Common Key for Visuospatial Execution and Attention in Parkinson’s Disease
Source: Diagnostics (Basel). 2021 Jun 6;11(6):1042. doi: 10.3390/diagnostics11061042 (PMC8229154; doi:10.3390/diagnostics11061042)
Supplement: Supplementary file 1 [file diagnostics-11-01042-s001.zip › diagnostics-1049118-SI/SupplementaryFiles/suppFigureLegend.pdf]

Supplementary Figure Legend

Supplementary Figure 1. Group differences in network scores.

Network scores in the PD group were significantly reduced in the anterior cingulate (IC4, $p = 0.02$), cerebellar lobule VII (IC36, $p = 0.04$) or superior temporal gyrus (IC50, $p < 0.05$) compared with the Ctr group. In contrast, network scores in the PD group were significantly increased in cerebellar lobule VI (IC47, $p = 0.04$) or the inferior frontal gyrus (IC66, $p = 0.04$) compared with the Ctr group. AC, anterior cingulate; CB, cerebellum; CBN, cerebellar network; CCN, cognitive control network; Ctr, normal control; DMN, default mode network; IFG, inferior frontal gyrus; Lt, left; PD, Parkinson's disease; Rt, right; STG, superior temporal gyrus.
