# Supplementary material for: The Cerebellum Is a Common Key for Visuospatial Execution and Attention in Parkinson’s Disease
Source: Diagnostics (Basel). 2021 Jun 6;11(6):1042. doi: 10.3390/diagnostics11061042 (PMC8229154; doi:10.3390/diagnostics11061042)
Supplement: Supplementary file 1 [file diagnostics-11-01042-s001.zip › diagnostics-1049118-SI/SupplementaryFiles/suppTables_v2.pdf]

Supplementary TABLES

Supplementary Table 1. Summary of the independent components.

				MNI coordinates (mm)		
**Network**	**Number of IC**	**Region**	**BA/Lobule**	**x**	**y**	**z**
**DMN**	4	AC	32	0	28	−12
	6	PC	23	0	−56	22
	8	AC	24	0	26	18
	10	Prc	7	0	−66	54
	12	Prc	7	−2	−78	40
	15	MeFG	11	0	50	−8
	17	MeFG	8	0	48	46

	18	MeFG	10	0	64	10
	25	SFG	8	6	36	52
	60	AC	24	2	32	−6
	67	SFG	6	−4	12	58
**CCN**	11	STG	38	−36	10	−28
	20	ITG	21	−62	−4	−22
	22	STG	38	36	12	−28
	24	Uncus	20	34	2	−40
	28	IPL	40	−48	−36	38
	31	MTG	21	−66	−30	−18
	34	IPL	40	46	−56	50

37	MiFG	10	22	64	24
38	IFG	46	50	48	6
39	STG	22	62	−52	10
43	MiFG	8	30	32	46
44	STG	38	−42	2	−20
45	IPL	40	66	−34	28
48	MiFG	46	56	24	28
49	MiFG	10	−30	60	6
50	STG	38	−44	14	−10
51	MiFG	10	−34	60	16
56	SFG	8	−18	46	42

	57	MTG	21	68	−32	−14
	59	MFG	10	−44	52	0
	61	MFG	10	34	64	−8
	64	IPL	40	−50	−64	48
	66	IFG	46	−54	30	48
	71	STG	38	44	8	−20
**SMN**	1	PrG	4	62	−4	24
	5	SPL	7	−18	−60	68
	7	PoG	3	42	−26	64
	9	PrG	4	−40	−24	66
	35	MeFG	6	8	0	64

	40	MeFG	6	2	−10	54
	42	MeFG	6	−2	−30	70
**VIS**	14	Cuneus	30	−12	−60	8
	30	Cuneus	19	−28	−86	34
	46	LG	17	10	−98	−4
	54	ITG	37	54	−72	−4
	62	MTG	19	−56	−66	16
	63	Cuneus	18	4	−82	16
**AUD**	23	STG	42	−60	−26	8
**CBN**	36	Cerebellum	VII	−30	−84	−46
	47	Cerebellum	VI, VII, VIII	−50	−66	−24

	65	Cerebellum	VI, VII	−34	−86	−26
**SCN**	3	CN	NA	−6	12	−2
	33	Putamen	NA	−24	2	−8

Abbreviations: AC, anterior cingulate; AG, angular gyrus; AUD, auditory network; BA, Brodmann area; CBN, cerebellar network; CCN, cognitive control network; CI, confidence interval; CN, caudate nucleus; DMN, default mode network; HY, Hoehn-Yahr stage; IFG, inferior frontal gyrus; IPL, inferior parietal lobule; ITG, inferior temporal gyrus; LED, levodopa equivalent dose; LG, lingual gyrus; MeFG, medial frontal gyrus; MiFG, middle frontal gyrus; MTG, middle temporal gyrus; NA, not available; PC, posterior cingulate; PoG, postcentral gyrus; Prc, precuneus; PrG, precentral gyrus; SCN, sub-cortical network; SFG, superior frontal gyrus; SMN, somatomotor network; SPL, superior parietal lobule; STG, superior temporal gyrus; VIS, visual network.
