# Supplementary figures and images for: The Cerebellum Is a Common Key for Visuospatial Execution and Attention in Parkinson’s Disease
Source: Diagnostics (Basel). 2021 Jun 6;11(6):1042. doi: 10.3390/diagnostics11061042 (PMC8229154; doi:10.3390/diagnostics11061042)

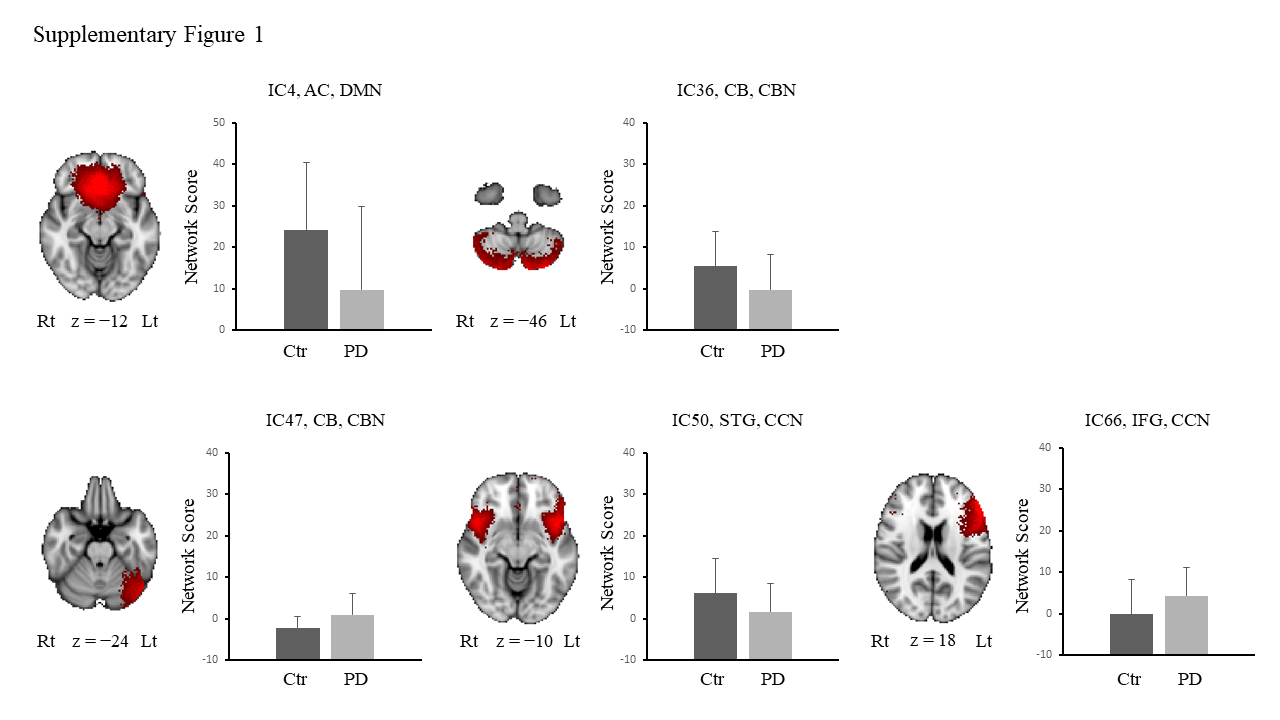

Supplement: Supplementary file 1 [file diagnostics-11-01042-s001.zip › diagnostics-1049118-SI/SupplementaryFiles/SupplementaryFigure1.tif]
